# In Vivo Hypoglycaemic Effect and Inhibitory Mechanism of the Branch Bark Extract of the Mulberry on STZ-Induced Diabetic Mice

**DOI:** 10.1155/2014/614265

**Published:** 2014-08-06

**Authors:** Hua-Yu Liu, Meng Fang, Yu-Qing Zhang

**Affiliations:** Silk Biotechnology Laboratory, School of Biology and Basic Medical Sciences, Soochow University, RM 702-2303, No. 199, Renai Road, Dushuhu Higher Edu. Town, Suzhou 215123, China

## Abstract

Branch bark extract (BBE) derived from the mulberry cultivar Husang 32 (*Morus multicaulis* L.) with aqueous alcohol solution has been investigated as an inhibitor of *α*-glycosidase *in vitro*. Mulberry BBE was orally administered to STZ-induced diabetic mice for three weeks, and it improved the weight gain and ameliorated the swelling of liver and kidney in diabetic mice. Obviously, mulberry BBE not only can reduce the abnormally elevated levels of serum insulin and ameliorate insulin resistance induced by STZ, but also it regulates dyslipidemia in diabetic mice. To understand this therapeutic effect and the regulatory mechanisms of BBE in diabetic mice, a qRT-PCR experiment was performed, indicating that the mulberry BBE can regulate the mRNA expression of glycometabolism genes in diabetic mice, including glucose-6-phosphatase (G6Pase), glucokinase (GCK), and phosphoenolpyruvate carboxykinase (PEPCK), thereby regulating sugar metabolism and reducing the blood glucose level in diabetic mice. The mulberry BBE can increase the mRNA expression of the genes Ins1, Ins2 and pancreatic duodenal homeobox-1 (PDX-1) and may decrease the insulin resistance in diabetic mice. Those results provide an important basis for making the best use of mulberry branch resources and producing biomedical drugs with added value.

## 1. Introduction

Mulberry (*Morus L.*) is a perennial woody economic plant. Mulberry is widely cultivated in China, India, Brazil, North Korea, and other countries. Mulberry leaf is mainly used as food for silkworms, and more than ten million tons of mulberry branches are harvested in China every year [1]. Most of the mulberry branch is wasted as agricultural trash or firewood. Only a small portion of the mulberry branches was used, as culture matrix for edible funguses and as composite wood materials, among other functions [2, 3]. However, the mulberry leaf (*Folium mori*), mulberry fruit (*Fructus mori*), mulberry branch (*Ramulus mori*), and mulberry root (*Cortex mori*) have been traditional Chinese medicinal herbs. Therefore, the development of new products and uses for mulberry branches has gained increasing attention. 

Modern pharmacological studies have demonstrated that the extracts of root bark, leaf, and branch bark of mulberry all have a variety of biological activities and pharmacological effects, such as hypolipidemic [4–6], antioxidation [7], antitumor [8], antibacterial, and anti-inflammatory ones [9–11]. In recent years, with the development of modern scientific technology, some studies have shown that there were evident differences in the chemical composition, pharmacological effects, and other health-related functions among the four parts of mulberry. Mulberry root bark contains many types of antioxidants, including flavonoids, guangsangons A-E [12], guangsangons K-N [13], and astragalus [14] (similar to resveratrol), and polysaccharides [15]. Most of these compounds have antibacterial and anti-inflammatory effects [16–18]. Mulberry branch extract can inhibit melanin formation and induce whitening [19, 20]. Mulberry branch alcoholic extract can also protect the kidney of hyperuricemic mice [21]. In addition,* cis*-mulberroside A in the branch extracts protects against alcohol-induced liver injury [22] and nerve damage [23].

Diabetes is a group of metabolic diseases caused by reduced or absent insulin secretion and it makes cells less sensitive to insulin. There are some structural defects in insulin itself that lead to chronic hyperglycemia and metabolic disorders of carbohydrate, protein, and fat. In traditional Chinese medicine, diabetes belongs to the consumptive thirst diseases. Mulberry leaves were found to have antidiabetic function in ancient Chinese medicine. The “Compendium of Materia Medica” shows the mulberry leaf as one of the set of drugs commonly used as antidiabetics [24]. “Bencao Tujing” recorded that the mulberry branch bark can help with digestion, diuresis, and dry mouth [25]. Combining mulberry branch bark with other traditional Chinese medicinals has a good hypoglycaemic effect [26]. In 1998, Nojima et al. isolated an extract from mulberry leaves and found that 1-deoxynojirimycin (DNJ) has an antidiabetic effect on STZ-diabetic mice [27]. DNJ can reduce the blood glucose level in alloxan-induced diabetic mice by activating PDX-1 (pancreatic duodenal homeobox-1)/insulin-1 signalling pathways and regulating the levels of glucokinase, pyruvate carboxylase, enol phosphate, and glucose 6-phosphate [18]. In the bark extract of mulberry root, flavonoids also have good hypoglycaemic activity that can reduce the insulin resistance of KK-Ay mice [28–30]. Mulberry latex can significantly reduce blood glucose through its very high concentrations of DNJ (dry weight 4.5%) [31]. Mulberry branch bark contains more DNJ than the leaves or the root bark [32]. Recent studies have shown that mulberry branch bark extract can significantly reduce blood glucose levels. The branch bark extract (BBE) can be used as a novel alpha-glucosidase inhibitor [33], with an effect quite similar to that of acarbose on diabetes complications and hyperglycemia syndrome in alloxan rats [34]. Continuous oral administration of the aqueous extract of mulberry branch bark for 15 days can significantly reduce the indices of hyperglycemia syndrome and improve blood lipids, diabetes, and kidney disease in hyperglycemic rats [33]. A recent investigation by our group showed that mulberry BBE can significantly inhibit sucrase and maltase* in vitro*, for IC_50_ values of sucrase and maltase of 6.5 *μ*g/mL and 0.25 *μ*g/mL, respectively [35], and the BBE also has a hypolipidemic effect [6]. The polysaccharide extract of white mulberry branch bark also can reduce the level of blood glucose in STZ-diabetic mice, and this effect is mediated by blocking inflammation and diminishing oxidative stress [36]. The hypoglycaemic substances in the mulberry branch bark involve not only alkaloids, such as DNJ, but also flavones, flavonoids, polysaccharides, and other compounds.

This work investigated the indices of hyperglycemia syndrome in STZ-induced diabetic mice treated orally with the alcohol-water extract of mulberry branch bark. Meanwhile, the therapeutic effect on the diabetic mice treated with the mulberry extract and the related hypoglycaemic mechanism was also investigated with qRT-PCR.

## 2. Materials and Methods

### 2.1. Materials

The branches of the mulberry cultivar HuSang 32 from* Morus multicaulis* L were collected from the Mulberry Garden of Soochow University, Suzhou, China, in November 2011. The ICR male mice (18–22 g BW) of clean grade were obtained from the Experimental Animal Centre of Soochow University. Rat intestinal enzymes and STZ were purchased from Sigma-Aldrich Fine Chemicals (USA), and PNPG was obtained from Calbiochem (Germany). Acetonitrile (HPLC grade) was purchased from Fisher Scientific (USA). Methanol (HPLC grade) was obtained from Spectrum Co. (USA). All other solvents and chemicals used were of analytical grade.

### 2.2. Sample Preparation

The mulberry BBE was prepared as reported by Zhang et al. [37]. The bark was peeled from mulberry branches, dried at 100°C for 2 h, and then pulverised. The raw bark powder was extracted for 1–48 hours with 10–90% ethanol solution at 80°C–100°C and then treated with papain (2%), shaken in the enzyme solution for 2 hours at 60°C, and extracted by the savage method (chloroform: n-butanol 4 : 1) several times to remove the miscellaneous protein. Then, the extracted solution was treated with active carbon to remove some of the pigments. Finally, the extract was made into powder with a spraying dryer.

### 2.3. Test of *α*-Glucosidase Inhibition

The inhibition of *α*-glucosidase was assayed with the method described by Yatsunami et al. (2003) with a slight modification [38]. Rat gut enzymes (100 mg/mL in 0.9% NaCl) were treated with ultrasound 3× for 1 min and then centrifuged at 3000 rpm for 30 min. The supernatant was diluted 1 : 4 with distilled water and stored at 4°C for later use. Then, 80 *μ*L of BBE solution (sample group) or phosphate buffer (control group) and 50 *μ*L of *α*-PNPG were added to a test tube. After incubation for 5 min at 37°C, 50 *μ*L of *α*-glucosidase from the rat intestine solution was added, and the mixture was blended. The mixture was then incubated for 15 min at 37°C. To stop the reaction, 140 *μ*L of Na_2_CO_3 _(0.2 mol/L) was added. The reaction mixture absorbance at 405 nm was measured in a 96-well microplate. The percentage of *α*-glucosidase inhibition in the sample was calculated using the following formula: inhibition rate (%) = (AC⁡−ACB)−(AS − ASB)/(AC⁡−ACB) × 100 (where AS, AC, ASB, and ACB represent the absorbance of the sample, control, sample blank, and the control blank, resp.).

When the inhibition percentage of the *α*-glucosidase activity was 50%, the BBE sample concentration was defined as the IC_50_ value. Each sample was analysed three times.

### 2.4. Administration to STZ-Diabetic Mice

The male ICR mice were maintained under controlled conditions (18–25°C, 50–80% humidity, and a 12-hour light/dark cycle). A standard pellet diet and water were given ad libitum. The mice were reared for one week, and then STZ (100 mg/kg) was injected into the mice through caudal vein. To determine the level of fasting blood-glucose (FBG), 100 *μ*L of blood was collected through the caudal vein after 72 h, and the mice with FBG ≥7.8 mmol/L were selected as the diabetic model. Subsequently, the mice were randomly divided into five groups (10 mice/Group): control group, model group, and three experimental groups. The control group and model group were treated with daily oral gavage of normal saline. The experimental groups were treated with HuSang 32 mulberry BBE at doses of 50, 100, and 200 mg/kg*·*BW; the extract was administered by daily oral gavage.

The body weights of all the mice were recorded every three days. The blood glucose levels of the mice were monitored after they were fasted for 12 h. All mice were fed for 3 weeks. At the end of the feeding period, all the animals were fasted for 12 h. Then, blood was collected from the eye portal vein. Blood glucose concentrations were measured with a blood glucometer, and body weight was recorded from each mouse. Finally, all mice were anesthetised with sodium pentobarbital and euthanised by cervical decapitation. Their livers, kidneys, and pancreases were excised and weighed. 

Animal experiments are all abided by the rules of the international animal welfare committee requirements and regulations. The experiment has been ethically acceptable. All the experimental procedures were approved by the Committee on Animal Experimentation and Ethics of Soochow University [number of animal license SCXK2011-0007].

### 2.5. Serum Insulin Measurement

Plasma from the mice was allowed to rest for 1 h at room temperature. After blood clotting, it was centrifuged at 4,000 rpm for 15 min, and the supernatant was taken for analysis. The mouse serum insulin was determined following the insulin kit instructions. The kit reagent was incubated at room temperature for 0.5 h. Standard 1–Standard 5 were dissolved in 50 *μ*L distilled water and blended before first use. Serum samples (50 *μ*L) were added to an ELISA plate with 50 *μ*L HRP-conjugate added each well (except the control well). Samples were fully blended and incubated for 120 min at 37°C, and then the liquid was discarded and the board was patted dry after three washes. Reagent A (50 *μ*L) and reagent B (50 *μ*L) were added to each well. After blending, the reaction results were coloured in a dark place for 15 min at 37°C. Then, stop buffer (NH_2_SO_4_, 50 *μ*L) was added to each well, and the OD value was determined with an ELISA instrument at 450 nm.

### 2.6. Blood Lipid (TG, CHO, HDL, and LDL) Determination

Mouse plasma was allowed to rest for 1 h at room temperature for blood clotting and then centrifuged at 4,000 rpm for 15 min, and the supernatant was used for analyses. The total cholesterol and triglyceride levels were determined according to the kit instructions. The kit reagent was incubated at room temperature for 0.5 h. The kit was used to prepare the standard substance, which was added to the reaction solution in turn. This reaction was stopped with H_2_SO_4_ stop buffer, and the OD value was determined with an ELISA instrument at 450 nm. The levels of HDL and LDL were calculated.

### 2.7. The Preparation of Pathological Tissue

The liver and pancreas were quickly removed from the mice, washed with normal saline, and then dried, weighed, cut into small pieces of 1 × 0.5 × 0.2 cm, and fixed with 10% formalin. The tissue dehydration was performed with increasing concentrations of acetone. Then, the sample was cleared with xylene and embedded in wax. Slices of 3-mm thickness were cut on an HM340E microtome, stained with H&E, and imaged under an optical microscope.

### 2.8. Preparation of mRNA and cDNA

#### 2.8.1. Total RNA Extraction

1. Cut tissues into small pieces and place them in glass homogeniser. Then, add 1 mL TRIZOL per 50–100 mg tissue to lyse and mix thoroughly. Homogenise the tissue. Then, transfer the mixture into a new 1.5-mL centrifuge tube. Alternatively, grind tissues into powder in liquid nitrogen. Stand at 15–30°C for 5 min. Centrifuge at 12,000 rpm at 4°C for 10 min.
2. Phase separation:
  - stand the homogenate at 15~30°C for 5 min to lyse completely;
  - add 0.2 mL chloroform to 1 mL TRIZOL Reagent. Shake and stand at 15–30°C for 2-3 min;
  - centrifuge at 10,000 rpm at 4°C for 10 min, at which point the mixture is divided into three phases. The RNA is in the top aqueous phase.
3. Transfer the upper clear phase to a fresh tube, taking care not to disturb the interface. Add 0.5 mL of isopropanol per millilitre of the clear phase. Mix gently by inverting the tube several times.
4. Let it stand for 10 min at 15~30°C and then centrifuge it at 10,000 rpm at 4°C for 10 min. Collect the precipitated RNA by centrifugation at 10,000 g for 10 min at 4°C. Carefully decant the supernatant. Remove any remaining liquid with a pulled Pasteur pipette. Immediately resuspend the pellet (without drying) in 75% ethanol to wash by vortexing with isopropanol. Centrifuge it at 7, 500 rpm for 5 min at 4°C.
5. The supernatant was removed, and the RNA pellet was washed once with 75% ethanol. The pellet was then air-dried and dissolved in RNase-free water (20–50 *μ*L) and stored at −70°C.

#### 2.8.2. cDNA Synthesis

*(1) The Determination of RNA Content*. Concentration of total RNA was determined with a nanovolume spectrophotometer. Using 1 *μ*L RNase-free water as the control, the RNA concentrations of samples were determined at 230 nm, 260 nm, and 280 nm. The ratio of OD_260_/OD_280_ should be between 1.8 and 2.0, and the amount of total RNA (ng/*μ*L) was recorded.

*(2) Reverse Transcription Reaction System*. The RT reaction system was assembled in a 0.6-mL PCR tube on ice, according to Table 1; total RNA should be no more than 500 ng.

*(3) The Conditions of the Reverse Transcription Reaction Were as Follows*. The reaction system was set to 37°C for reverse transcription reactions, and the heating block was removed after 15 min. The reverse transcriptase was inactivated by heating to 85°C for 5 sec in a heating block. The cDNA from the reverse transcription reaction was stored at −20°C.

#### 2.8.3. Primer Design

The primers were designed based on the gene sequences of glucose-6-phosphatase, glucokinase, phosphoenolpyruvate, and *β*-actin (see Table 2). *β*-Actin was used as an internal control, and all the primers were synthesised by Sangon Biotech (Shanghai) Co., Ltd.

#### 2.8.4. qRT-PCR

To generate the standard curve, the cDNA was diluted to 1 : 10, 1 : 100, and 1 : 1000, and the reaction solutions were prepared according to Table 3. The SYBR PrimeScript TMRT-PCR kit was used according to the manufacturer's instructions. The reaction system capacity is 20 *μ*L. Reaction procedure: incubate, 50°C for 2 min; 95°C initial denaturation for 1 min; 45 cycles of the following: denature, 95°C for 15 s; anneal, 60°C for 31 s; elongate, 72°C for 30 s. Each sample was repeated 3 times. The real-time PCR data were analysed with Sequence Detection Software Version 1.3.1. The data were rectified using Jan H Schefe's method. Using the different concentrations of cDNA as template, the standard curves of the experimental genes and the internal reference gene were generated. The data were calculated according to the following formula: (*T*) = *a* × *lg*(copy) + *b*; values of *a* vary among the different genes and PCR fragments.

### 2.9. Determination of mRNA Levels of INS-1, INS-2, and PDX-1 in Pancreas

The primers were designed based on the gene sequences of insulin-1 (INS-1), insulin-2 (INS-2), and PDX-1 (see Table 4). *β*-Actin was used as an internal control, and all the primers were synthesised by Sangon Biotech (Shanghai) Co., Ltd. The methods for total RNA extraction and cDNA synthesis from pancreas use 2.4.7.1 and 2.4.7.2 as references, and qRT-PCR was performed according to 2.4.7.4. Sequence Detection Software Version 1.3.1 was used to analyse the qRT-PCR data. The data were rectified using Jan H. Schefe's method. Using the different concentrations of template cDNA, the standard curves of the experimental and internal reference genes were generated. The data were calculated with following formula: *C*(*T*) = *a* × *lg*(copy) + *b*, with *a*-values varying among the different genes and PCR fragments.

### 2.10. Recording and Analysis of Experimental Data

The experimental data were recorded accurately and analysed with Origin 7.5 software. Results reported as mean ± SD, ANOVA, were used to evaluate the difference between multiple groups, with *P* < 0.05 considered as significant and *P* < 0.01 as very significant.

## 3. Results

### 3.1. The Effect of BBE on Weight of Diabetic Mice

**Figure 1:**
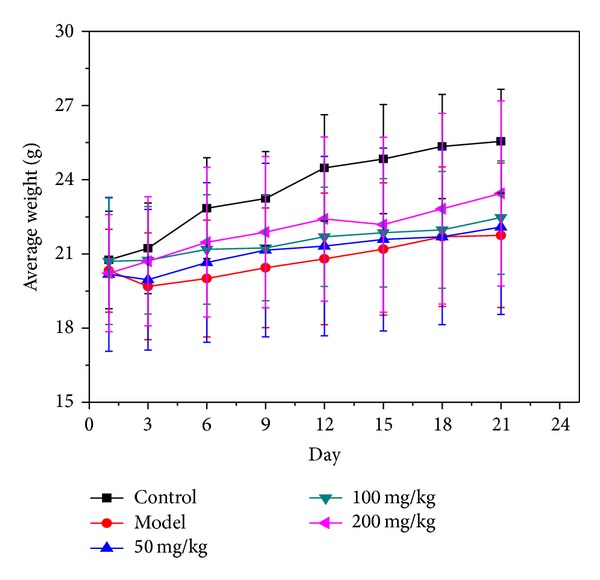
The effect of BBE on the body weight of STZ-induced diabetic mice.

During the test, all of the mice were in the growth phase. Compared with normal controls, the STZ-induced diabetic mice showed polyuria, polyphagia, and weight loss. Weight growth was slow in diabetes model mice; they lost weight during the first three days. With 50 and 100 mg/kg*·*BW BBE treatment, the weight loss was slower and their weights remained higher than that of the model group. Compared with the diabetes model group, the weight of the mice in the high-dose group increased (Figure 1). As shown in Table 5, the liver weight and kidney weight of diabetes model mice were significantly higher than normal control mice, but there was no obvious change in spleen weight. In the three treatment groups (treatment with 50 mg/kg*·*BW, 100 mg/kg*·*BW, and 200 mg/kg*·*BW of Husang 32 mulberry BBE), the liver and kidney swellings were somewhat alleviated, most obviously in the high-dose group, and the weight index was close to the normal control group. These results show that treatment with BBE can reduce the enlargement of liver and kidney in STZ-diabetic mice.

### 3.2. Effect of BBE on the Blood Glucose Level of STZ-Diabetic Mice

**Figure 2:**
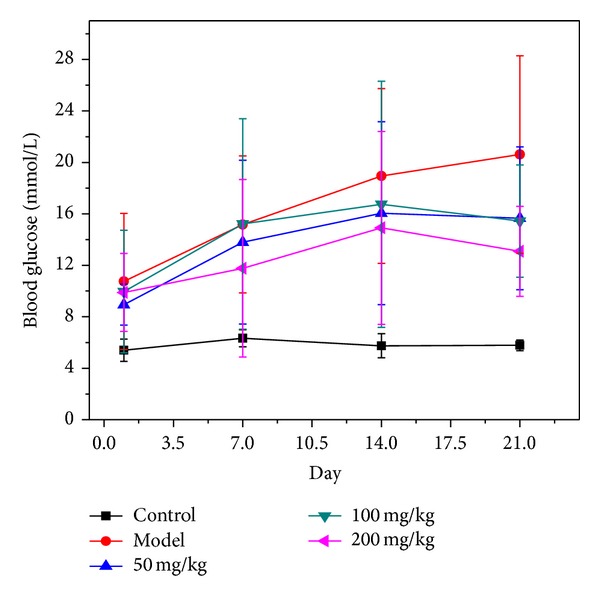
The effect of HuSang 32 branch bark extract on STZ-diabetic mice blood glucose level.

After three weeks of gavage, as shown in Figure 2, the fasting glucose level of the normal control mice was maintained at approximately 5.7, with no significant changes before or after the experiment. The average glucose level in STZ-diabetic mice continued to rise, and it also showed significant changes between before and after the experiment when compared with the normal control group. The FBG levels of mice treated with 50 or 100 mg/kg*·*BW BBE showed the same varying trend, which declined during the third week. In the high-dose (200 mg/kg*·*BW) group, FBG rose gently compared with the model group, and its FBG decreased in the third week and returned to normal. These results show that FBG was reduced depending on the time and dose of BBE treatment in STZ-diabetic mice.

### 3.3. Effects of BBE on Serum Insulin in Diabetic Mice

**Figure 3:**
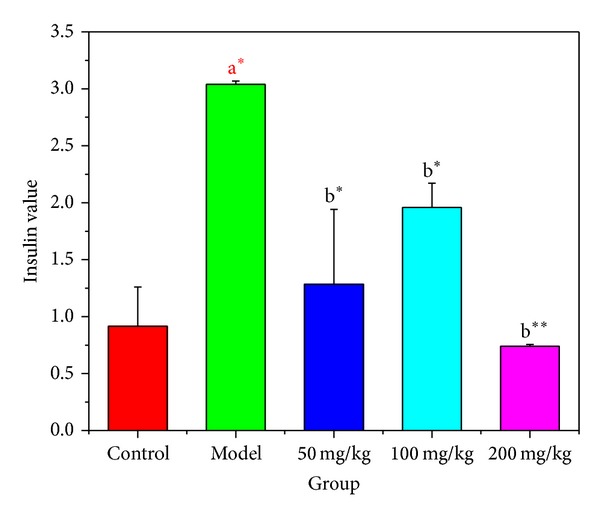
The effect of HuSang 32 branch bark extract on diabetic mice insulin level. A^∗^, compared with normal control group, *P* < 0.05, significant difference; b^∗^ compared with model group, *P* < 0.05, significant difference; b^∗∗^, compared with model group, *P* < 0.01, very significant.

In insulin resistance, insulin secretion is too high to maintain blood glucose stability because the uptake and utilization of glucose has been reduced. Too much insulin leads to reductions in the sensitivity of insulin receptors, and the ability to dissolve glucose declines. STZ can induce insulin resistance. As shown in Figure 3, the serum insulin levels in diabetic model mice were significantly higher than that in the normal control group (*P* < 0.05). With different doses of BBE treatment for 3 weeks, the serum insulin level of the diabetes mice improved significantly (*P* < 0.05). The serum insulin levels in the 200 mg/kg treatment group decreased dramatically compared with the model group, to a very significant degree (*P* < 0.01). The serum insulin levels in the 200 mg/kg BBE treatment group approached those of the normal control group, with no significant difference (*P* > 0.05). These results show that treatment with the HuSang 32 mulberry BBE can reduce the serum insulin levels effectively and improve insulin resistance in STZ-diabetic mice.

### 3.4. Effects of BBE on Serum Lipid Level in STZ-Diabetic Mice

Diabetes can lead to complications such as lipid and protein metabolism disorders. As shown in Table 6, TG levels in diabetic mice were significantly higher than those of normal control group mice (*P* < 0.05). After 3 weeks of treatment with different doses of BBE, the TG level of the diabetic mice had improved significantly. The TG levels of the 50 and 100 mg/kg BBE treatment groups were significantly lower than those of model group mice (*P* < 0.05). The TG levels of the treatment groups were lower than those of model group. It indicated that the BBE had hypolipidemic effect, which is very similar to the results reported by Liu and Zhu [32]. The LDL-C levels in diabetic mice were significantly higher than those in the normal control group (*P* < 0.05). After 3 weeks of treatment with different doses of BBE, the LDL-C levels of the diabetic mice had improved significantly. The LDL-C levels of the 100 mg/kg BBE treatment group declined dramatically and approached those of the normal control group, with no significant difference (*P* > 0.05). The CHOL levels in diabetic mice were significantly higher than those in the normal control group (*P* < 0.05). Administration of different doses of BBE for 3 weeks did not significantly improve the CHOL level. The CHOL levels of the 100 mg/kg treatment group showed no significant difference compared with the normal control group (*P* > 0.05). These results show that the HuSang 32 mulberry BBE can reduce the serum CHOL levels in STZ-diabetic mice. The HDL-C levels in diabetic mice showed no significant difference from the normal control group. Administration of different doses of BBE for 3 weeks did not significantly change the HDL-C level. These results show that the administration of BBE cannot improve the HDL-C level in STZ-diabetic mice.

### 3.5. Effects of BBE on Pathologic Tissue in the Liver of STZ-Diabetic Mice

**Figure 4:**
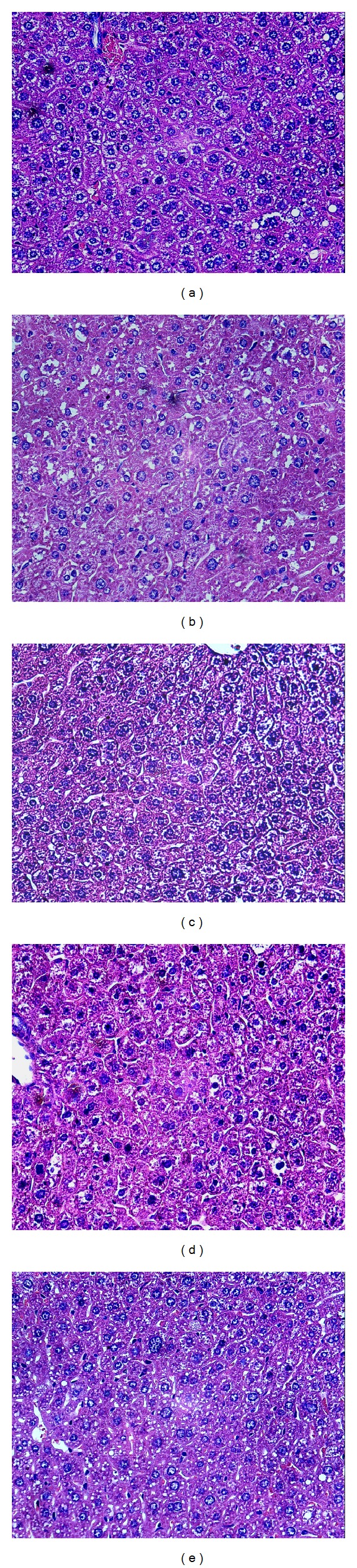
Effects of BBE on pathologic tissue in the livers of STZ-diabetic mice. (a) Normal group; (b) diabetes model control group; (c), (d), and (e) STZ-induced diabetic mice treated with 50, 100, and 200 mg/kg*·*BW of BBE, respectively.

The liver is a vital organ in glucose metabolism. Pathological changes in the mouse liver tissue are shown in Figure 4. Normal mouse liver cells are arranged compactly, and the hepatic lobules are clearly visible (Figure 4(a)). Liver cells in diabetic mice lost their normal structure, showing swelling, granular degeneration, local necrosis, and focal necrosis, and cells have become pyknotic, karyorrhexic, and karyolytic. The liver cytoplasm was filled with lipid droplets of various sizes and showed fatty degeneration. The central vein has mild congestion, and there is minor infiltration by perivascular inflammatory cells (Figure 4(b)). With the administration of different doses of BBE for 3 weeks, liver cell necrosis and liver damage were reduced in each treatment group. In the 200 mg/kg BBE treatment group, the liver cells lined up tightly, the liver cells boundaries were visible, and liver cell particles were only slightly deformed (Figure 4(e)).

### 3.6. Effects of BBE on Pathologic Tissue in the Pancreas of STZ-Diabetic Mice

**Figure 5:**
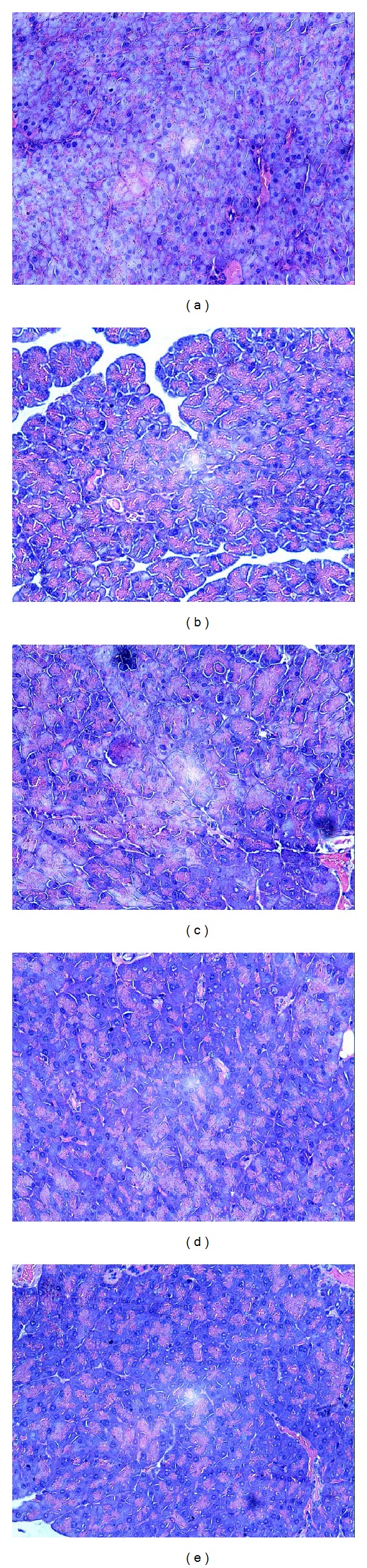
Effects of BBE on pathologic tissue in the pancreas of STZ-induced diabetic mice. (a) Normal group; (b) diabetes model control group; (c), (d), and (e) STZ-induced diabetic mice treated with 50, 100, and 200 mg/kg*·*BW of BBE, respectively.

The pancreas can secrete insulin and is an important organ for regulating blood glucose.  Figure 5 shows the pathological changes in the pancreas tissue of experimental mice. In normal mice, pancreatic cells are arranged compactly, and the cell nuclei are clearly visible. In diabetic mice, the pancreatic cells lost their normal structure, showing swelling, granular degeneration, local necrosis, and focal necrosis, and cells have become pyknotic, karyorrhexic, and karyolytic. With the addition of different doses of BBE for 3 weeks, pancreatic cell necrosis and pancreatic damage were reduced in each treatment group. In the 100 mg/kg BBE treatment group, mice pancreatic cells were lined up tightly, and pancreatic cells particles were only slightly deformed (Figure 5(e)).

### 3.7. Effects of BBE on the mRNA Expression of Key Enzymes in Gluconeogenesis

The liver is a major organ in the regulation of glucose metabolism. Glycogen use and release are dependent on 6-phosphoric acid enzymatic synthesis and hydrolysis, and these reactions are catalysed by glucokinase (GCK) and glucose-6-phosphatase (G6Pase). GCK is the first rate-limiting enzyme of glycolysis; it catalyses glucose into glucose 6-phosphoric acid. GCK is also required for glycogen synthesis. G6Pase is a key enzyme of gluconeogenesis; it catalyses glucose 6-phosphate into glucose, and this reaction is the last step of glycometabolism and gluconeogenesis.

**Figure 6:**
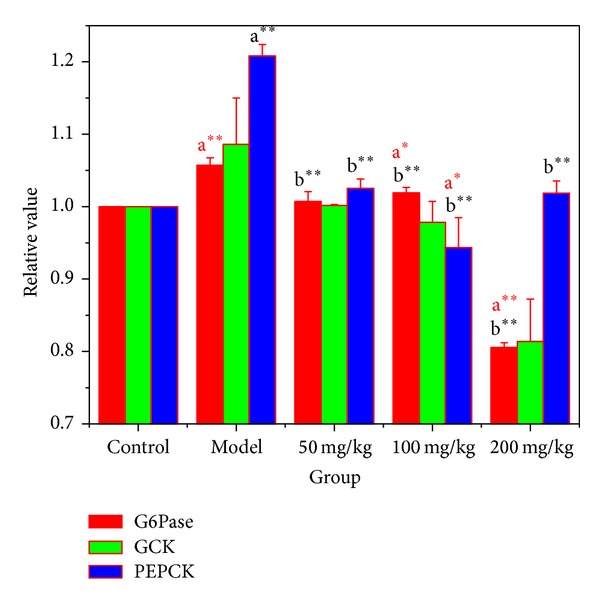
The effect of HuSang 32 branch bark extract on STZ-induced diabetic mice G6Pase, GCK, and PEPCK mRNA expression. a*, compared with normal control group, *P* < 0.05, significant difference; a^∗∗^, compared with normal control group, *P* < 0.01, very significant; b^∗∗^, compared with model group, *P* < 0.01, very significant.

As shown in Figure 6, when compared with the normal mice, the G6Pase mRNA expression in STZ-diabetic mice liver was significantly upregulated (*P* < 0.01). After 3 weeks of treatment with 50, 100, or 200 mg/kg*·*BW of BBE, the G6Pase mRNA expression in liver decreased below that in the diabetic model group, very significantly (*P* < 0.01). The G6Pase mRNA expression in liver decreased with the 200 mg/kg*·*BW BBE treatment.

As shown in Figure 6, compared with the normal mice, STZ-diabetic mice showed upregulated GCK mRNA expression in liver, with no significant difference. After 3 weeks of treatment with 50, 100, or 200 mg/kg*·*BW of BBE, the GCK mRNA expression in liver decreased. Liver GCK mRNA expression in the 200 mg/kg*·*BW BBE treatment group was very significantly decreased, below the level in the diabetic model group.

As shown in Figure 6, compared with the normal mice, the phosphoenolpyruvate carboxykinase (PEPCK) mRNA expression in STZ-diabetic mice liver was very significantly upregulated (*P* < 0.01). After 3 weeks of treatment with 50, 100, or 200 mg/kg*·*BW of BBE, the PEPCK mRNA expression in liver decreased very significantly, below that in the diabetic model group (*P* < 0.01). The PEPCK mRNA expression in liver in the 100 mg/kg*·*BW BBE treatment group was significantly decreased compared to that in normal mice (*P* < 0.01).

### 3.8. Effects of BBE on mRNA Expression of Mouse Ins1 and Ins2

**Figure 7:**
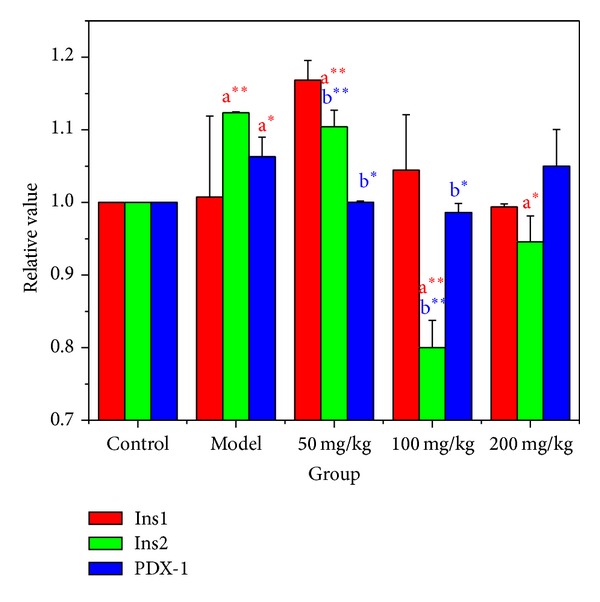
The effect of HuSang 32 BBE on STZ-induced diabetic mice Ins1, Ins2, and PDX-1 mRNA expression. a^∗^, compared with normal control group, *P* < 0.05, significant difference; a^∗∗^, compared with normal control group, *P* < 0.01, very significant; b^∗^, compared with model group, *P* < 0.05, significant difference; b∗∗, compared with model group, *P* < 0.01, very significant.

According to Figure 7, compared with the normal mice, the Ins1 mRNA expression in STZ-diabetic mice pancreas was nonsignificantly upregulated. After 3 weeks of treatment with 50, 100, or 200 mg/kg*·*BW of BBE, the Ins1 mRNA expression in pancreas decreased very significantly compared to that in normal mice. The Ins1 mRNA expression in mouse pancreas treated with 50 mg/kg*·*BW BBE was significantly upregulated compared to normal mice (*P* < 0.01).

As shown in Figure 7, compared with the normal mice, the Ins2 mRNA expression of STZ-diabetic mice in pancreas was very significantly upregulated. After 3 weeks of treatment with 50, 100, or 200 mg/kg*·*BW of BBE, the Ins2 mRNA expression in pancreas decreased significantly compared to that in normal mice. The Ins2 mRNA expression in mouse pancreas treated with 100 mg/kg*·*BW BBE was significantly decreased compared to normal mice.

As shown in Figure 7, compared with the normal mice, the PDX-1 mRNA expression in the pancreas of STZ-diabetic mice was significantly upregulated. After 3 weeks of treatment with 50, 100, or 200 mg/kg*·*BW of BBE, the PDX-1 mRNA expression in pancreas decreased significantly compared to the model group mice. The PDX-1 mRNA expression in pancreas treated with 200 mg/kg*·*BW BBE was not significantly different from that in normal mice.

## 4. Discussion

In recent years, with the rise of green drugs, many studies have been devoted to searching for safe and effective diabetes drugs from natural materials. The mulberry BBE has a hypoglycaemic effect that has been approved by the FDA of China and also has been clinically used [33]. Li et al. investigated the role of 1-deoxynojirimycin (DNJ) on glucose absorption and metabolism in normal and diabetic mice [39]. Of three parts of the mulberry, the mulberry leaf, mulberry branch phloem, and xylem, Liu et al. discovered that the DNJ level is highest in mulberry branch phloem (4.956 mg/g) [32]. Wang et al. investigated the biological activities of the branch bark ethanol extract in the mulberry (*Morus multicaulis* L.). The major components of the extract were the flavonoids, phenols, saccharides, and flavonoids glycosides. The branch bark extract has a good DPPH radical and can decrease the postprandial hyperglycemia of the type 2 diabetic mice significantly [40]. Several studies have been performed to evaluate the hypoglycaemic effect and antioxidation of mulberry branch bark extract [41, 42]. However, very little research in those areas has focused on the Husang 32 mulberry. This paper used the Husang 32 mulberry branch bark as an experimental material. The mulberry BBE was acquired via extraction procedures, and we have published a preliminary study of glucosidase inhibitors and explore the effect of mulberry BBE on STZ-diabetic mice. STZ not only destructs the part function of islet beta cells so that glucose metabolism disorders, but also induces insulin resistance, which results in the increase of insulin level in blood [43]. The experimental results show that the BBE can reduce hyperglycemia and improve abnormal blood lipids and insulin resistance in STZ-diabetic mice.

Most carbohydrates are glycoconjugates, and their digestion in the gut entails several sequential enzymatic reactions. The results show that the Husang 32 mulberry BBE can inhibit *α*-glycosidase and reduce postprandial blood glucose. The liver is the major organ for regulating glucose metabolism. In diabetes, insulin secretion is insufficient or there is insulin resistance, which can reduce the glucose utilisation and glycogen synthesis, inducing abnormal increases in gluconeogenesis [43]. Mulberry BBE may regulate key genes of glycometabolism in the liver to reduce gluconeogenesis and increase glycogen synthesis, so that blood glucose is reduced.

The efficiency of glucose uptake and utilisation by the promotion of insulin have dropped because it has many causes. The body secretes insulin excessively with compensation, to maintain blood glucose homeostasis, but it can lead to hyperinsulinaemia. If insulin is excessive, insulin receptor sensitivity can be reduced, thereby reducing the ability to process glucose that is called non-insulin-dependent diabetes mellitus. BBE can reduce the insulin resistance and may regulate genes that can regulate the insulin secretion and the effects on STZ-diabetic mice.

In conclusion, treatment with Husang 32 mulberry BBE can regulate carbohydrate absorption, the body's antioxidant ability, glucose metabolism, and insulin secretion. BBE can reduce the FBG in STZ-diabetic mice. This work shows that the Husang 32 mulberry BBE has preventive and treatment effects in STZ-diabetic mice. Although the BBE has many health benefits, it is a mixture that includes many components. Further studies are necessary to isolate individual components and characterise the beneficial biological properties observed in our study.

## 5. Conclusions

**C**ompared with the model group, BBE treatment can improve the weight gain and enlargement of the spleen and liver in STZ-diabetic mice. The extract can reduce serum insulin and improve insulin resistance in STZ-diabetic mice. BBE can increase the mRNA expression of the genes G6Pase, GCK, and PEPCK relative to the glucose metabolism in liver and genes Ins1, Ins2, and PDX-1 relative to insulin secretion, which indicates that HuSang 32 mulberry BBE may regulate insulin secretion and reduce insulin resistance in STZ-diabetic mice.

## Figures and Tables

**Table 1:** Reverse transcription reaction system.

Reagent	Quantity
5× PrimeScript Buffer (for real time)	2.0 *μ*L
PrimeScript RT Enzyme Mix I	0.5 *μ*L
Oligo dT Primer (50 *μ*M)	0.5 *μ*L
Random 6 mers (100 *μ*M)	0.5 *μ*L
Total RNA	X
RNase Free dH2O	UP TO 10 *μ*L

**Table 2:** mRNA primer design for the key metabolic enzymes in liver.

Target gene	(5′ to 3′) Primer sequences	Product length
G6Pas_F	CACCGACTACTACAGCAACAGC	209
G6Pas_R	AGAATCCCAACCACAAGATGAC	

GCK_F	CTTCACCTTCTCCTTCCCTGTAA	145
GCK_R	AAAGTCCCCTCTCCTCTTGATAG	

PEPCK_F	AGTCATCATCACCCAAGAGC	154
PEPCK_R	TGGGATGACATACATGGTGC	

*β*-actin-F	CAGCCTTCCTTCTTGGGTAT	91
*β*-actin-R	GGTCTTTACGGATGTCAACG	

**Table 3:** qRT-PCR reaction system.

Reagent	Usage
SYBR Premix Ex Taq	10.0 *μ*L
Rox	0.4 *μ*L
PCR Primer	0.8 *μ*L
DNA template	2.0 *μ*L
dH2O	6.8 *μ*L

**Table 4:** mRNA primer design for INS-1, INS-2, and PDX-1 in pancreas.

Target gene	Primer sequences (5′ to 3′)	The length of the product
Insulin-1_F	GTGATAAAACCCTGACAAGAGCA	141
Insulin-1_R	TGTGGGGATAATAGGAGCAGTT	

Insulin-2_F	CAAGTGGCACAACTGGAGCT	110
Insulin-2_R	CTGGTGCAGCACTGATCTAC	

Pdx-1_F	AGTGGGCAGGAGGTGCTTAC	186
Pdx-1_R	GGAACCAGATTTTGATGTGTCTCT	

*β*-Actin-F	CAGCCTTCCTTCTTGGGTAT	91
*β*-Actin-R	GGTCTTTACGGATGTCAACG	

**Table 5:** The effect of BBE on the tissue weight of STZ-induced diabetic mice.

Experimental group	Gavage dose (mg/kg)	Liver	Spleen	Kidney
Control	—	0.870 ± 0.1290	0.086 ± 0.0208	0.333 ± 0.0088
Model	—	1.27 ± 0.0378∗	0.080 ± 0.0265	0.373 ± 0.0328∗
Low dose	50	1.11 ± 0.0503∗	0.073 ± 0.0058	0.323 ± 0.0321
Middle dose	100	1.09 ± 0.1531	0.100 ± 0.0173	0.343 ± 0.0404
High dose	200	1.07 ± 0.1415	0.083 ± 0.0252	0.333 ± 0.0115

*Significant difference compared with the control group (*P* < 0.05).

**Table 6:** Effects of BBE on serum lipid levels in STZ-diabetic mice.

Serum lipid	TG (mmol/L)	CHOL (mmol/L)	HDL-C (mmol/L)	LDL-C (mmol/L)
Control group	3.326 ± 0.6165	3.566 ± 0.3110	2.526 ± 0.1718	0.224 ± 0.0439
Model group	3.758 ± 0.4121∗	4.112 ± 0.4194∗	2.740 ± 0.2889	0.392 ± 0.0576∗∗
50 mg/kg group	2.635 ± 1.1189^#^	4.426 ± 0.5919∗	2.940 ± 0.3939	0.420 ± 0.0994∗∗
100 mg/kg group	2.728 ± 0.4906^#^	3.610 ± 0.5516	2.440 ± 0.3136	0.313 ± 0.0924
200 mg/kg group	3.132 ± 0.3744	4.378 ± 0.5693∗	2.99 ± 0.3887∗	0.322 ± 0.0614∗

^∗ & ∗∗^Compared with normal control group with *P* < 0.05 and *P* < 0.01, respectively, significant differences, and very significant differences. ^#^Compared with model group with *P* < 0.05.
